# Association of Polyp Regression after Loading Phase with 12-Month Outcomes of Eyes with Polypoidal Choroidal Vasculopathy

**DOI:** 10.3390/ph17060687

**Published:** 2024-05-27

**Authors:** Misa Kimura, Yoichi Sakurada, Yoshiko Fukuda, Mio Matsubara, Yumi Kotoda, Yuka Kasai, Atsushi Sugiyama, Wataru Kikushima, Daphne Viel Tsuru, Kenji Kashiwagi

**Affiliations:** Department of Ophthalmology, Faculty of Medicine, University of Yamanashi, Shimokato 1110, Chuo 409-3821, Yamanashi, Japan; smisa@yamanashi.ac.jp (M.K.); ysugiyama@yamanashi.ac.jp (Y.F.); miom@yamanashi.ac.jp (M.M.); ykanai@yamanashi.ac.jp (Y.K.); kasayuka@yamanashi.ac.jp (Y.K.); asugiyama@yamanashi.ac.jp (A.S.); wkikushima@yamanashi.ac.jp (W.K.); dvctsuru-as@yamanashi.ac.jp (D.V.T.); kenjik@yamanashi.ac.jp (K.K.)

**Keywords:** polypoidal choroidal vasculopathy, aflibercept, brolucizumab, retreatment-free period, number of additional injections

## Abstract

Purpose: We compared 12-month outcomes of eyes with polypoidal choroidal vasculopathy (PCV) with or without complete regression of polyps observed one month after three monthly intravitreal administrations (loading phase) of aflibercept (2.0 mg/0.05 mL) or brolucizumab (6.0 mg/0.05 mL). Methods: All patients underwent indocyanine green angiography at both baseline and 3 months after initial injection and were followed up monthly with an as-needed regimen for up to 12 months. A total of 62 patients with PCV were included: 30 eyes were treated with brolucizumab, and 32 were treated with aflibercept. Eyes with complete regression of polyps (regression group) had significantly smaller maximum polyp diameter and were more frequently treated with brolucizumab than those without complete regression (non-regression) group. Results: Best corrected visual acuity was comparable between the two groups at 12 months. Although the 12-month retreatment-free proportion was comparable between the two groups (33.0% versus 27.0%, *p* = 0.59), a retreatment-free period was significantly longer in the regression group than in the non-regression group (8.3 ± 3.3 versus 6.5 ± 3.6 months, *p* = 0.022), and the number of additional injections was significantly fewer in the regression group than in the non-regression group (1.2 ± 1.2 versus 3.0 ± 2.6, *p* = 0.007). Conclusions: Complete regression of polyps observed after the initial phase possibly prolongs the retreatment-free period and reduces the number of additional injections irrespective of aflibercept or brolucizumab.

## 1. Introduction

Polypoidal choroidal vasculopathy (PCV)—a variant of type 1 macular neovascularization (MNV) secondary to exudative age-related macular degeneration (AMD)—accounts for almost half of advanced AMD observed in the Japanese population [1,2]. It is diagnosed as the presence of polypoidal lesions with or without a branching vascular network on indocyanine green angiography (ICGA), and some cases may have clinical characteristics of pachychoroid spectrum diseases distinct from drusen-driven MNV [3,4].

The current first-line treatment for PCV is intravitreal injection of vascular endothelial growth factor (VEGF) inhibitors, and a combination therapy regimen may include photodynamic therapy (PDT) [5,6]. Several studies have demonstrated that the regression rate of polyps varies depending on the kind of VEGF inhibitor used and whether it was combined with PDT. It has been reported that the regression of polyps after the loading phase was 40–75% for aflibercept, 57–93% for brolucizumab, and 70–85% for combination therapy with PDT [7,8,9,10,11,12,13,14,15,16,17]. 

The treatment efficacy for PCV is commonly evaluated by determining the presence or absence of exudation, including subretinal and intraretinal fluid on spectral domain/swept-source optical coherence tomography (SD/SS-OCT), and the regression rate of polyps observed on ICGA. But despite the common practice of evaluating treatment efficacy by the presence or absence of complete polyp regression on ICGA after a loading phase, there are only a few studies investigating the treatment outcomes in eyes with complete regression of polyps after the loading phase [18,19]. In this study, we investigated possible factors predictive of complete polyp regression after three monthly intravitreal injections of aflibercept (2.0 mg/0.05 mL) or brolucizumab (6.0 mg/0.05 mL). We compared 12-month treatment outcomes between eyes with or without complete polyp regression after the loading phase on ICGA with a pro re nata (PRN) regimen.

## 2. Results

### 2.1. Demographics and Clinical Characteristics

Table 1 shows the demographic and clinical characteristics of patients with PCV. Sixty-two (62) eyes with PCV met the inclusion criteria. Aflibercept (2.0 mg/0.05 mL) was administered to 32 eyes, while brolucizumab (6.0 mg/0.05 mL) was administered to 30 eyes. Anterior chamber inflammation was observed in one eye treated with brolucizumab, which resolved with the use of betamethasone 0.1% eye drops. Table 2 compares the baseline characteristics between the complete regression group and the non-complete regression group. The baseline maximum polyp size was significantly greater in the non-complete regression group than in the complete regression group. (293 ± 140 μm versus 457 ± 262 μm, *p* = 0.024). Complete regression was more frequently observed in the brolucizumab group (67%) compared with aflibercept (33%) (*p* = 0.001). Complete regression rates at the 3-month visit were 80% and 37.5% in the brolucizumab-treated group and the aflibercept-treated group, respectively (*p* < 0.001).

### 2.2. Differences in Best-Corrected Visual Acuity

**Figure 1:**
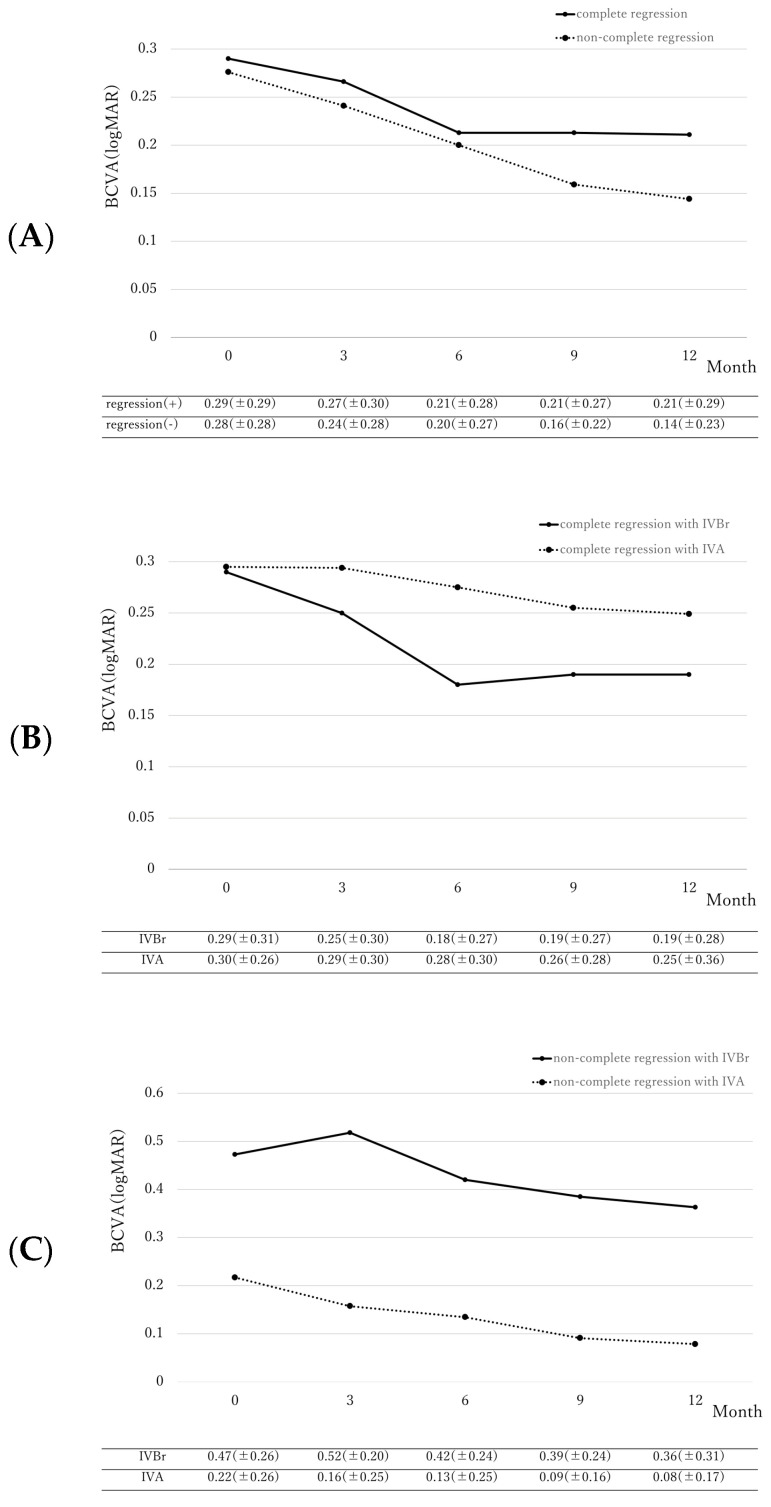
(**A**) Change of best-corrected visual acuity (BCVA) in eyes with complete regression of polyps (regression group) and without complete regression of polyps (non-regression group). BCVA significantly improved at 12 months in both the regression group (from 0.29 ± 0.29 logMAR to 0.21 ± 0.29 logMAR, *p* = 0.006). The extent of BCVA improvement was comparable between both groups (*p* = 0.39). (**B**) Change of BCVA in eyes with complete regression of polyps treated with aflibercept (IVA) or brolucizumab (IVBr). In complete regression eyes treated with brolucizumab, baseline BCVA significantly improved from 0.29 ± 0.31 logMAR to 0.19 ± 0.28 logMAR at 12 months (*p* = 0.004). However, BCVA in the complete regression group treated with aflibercept did not reach statistical significance (from 0.30 ± 0.26 logMAR to 0.25 ± 0.36 logMAR, *p* = 0.053). The extent of BCVA improvement was comparable between both groups (*p* = 0.59). (**C**) Change of BCVA in eyes with non-complete regression of polyps treated with aflibercept (IVA) or brolucizumab (IVBr). In the non-complete regression group, BCVA at 12 months of eyes treated with brolucizumab had a non-significant (*p* = 0.48) improvement from 0.47 ± 0.26 logMAR to 0.36 ± 0.31 logMAR while eyes treated with aflibercept showed significant improvement from 0.22 ± 0.26 logMAR to 0.08 ± 0.17 logMAR (*p* = 0.003). The extent of BCVA improvement was comparable between the two groups (*p* = 0.39).

BCVA changes are illustrated in Figure 1. BCVA significantly improved at 12 months in both the regression group (from 0.29 ± 0.29 logMAR to 0.21 ± 0.29 logMAR, *p* = 0.006) and the non-regression group (from 0.28 ± 0.28 logMAR to 0.14 ± 0.23 logMAR, *p* = 0.016). The extent of BCVA improvement was comparable between both groups (*p* = 0.39). In complete regression eyes treated with brolucizumab, baseline BCVA significantly improved from 0.29 ± 0.31 logMAR to 0.19 ± 0.28 logMAR at 12-months (*p* = 0.004). However, BCVA in the complete regression group treated with aflibercept did not reach statistical significance (from 0.30 ± 0.26 logMAR to 0.25 ± 0.36 logMAR, *p* = 0.053). The extent of BCVA improvement was comparable between both groups (*p* = 0.59). In the non-complete regression group, BCVA at 12 months of eyes treated with brolucizumab had a non-significant (*p* = 0.48) improvement from 0.47 ± 0.26 logMAR to 0.36 ± 0.31 logMAR while eyes treated with aflibercept showed significant improvement from 0.22 ± 0.26 logMAR to 0.08 ± 0.17 logMAR (*p* = 0.003). The extent of BCVA improvement was comparable between the two groups (*p* = 0.39).

### 2.3. Retreatment-Free Period

**Figure 2:**
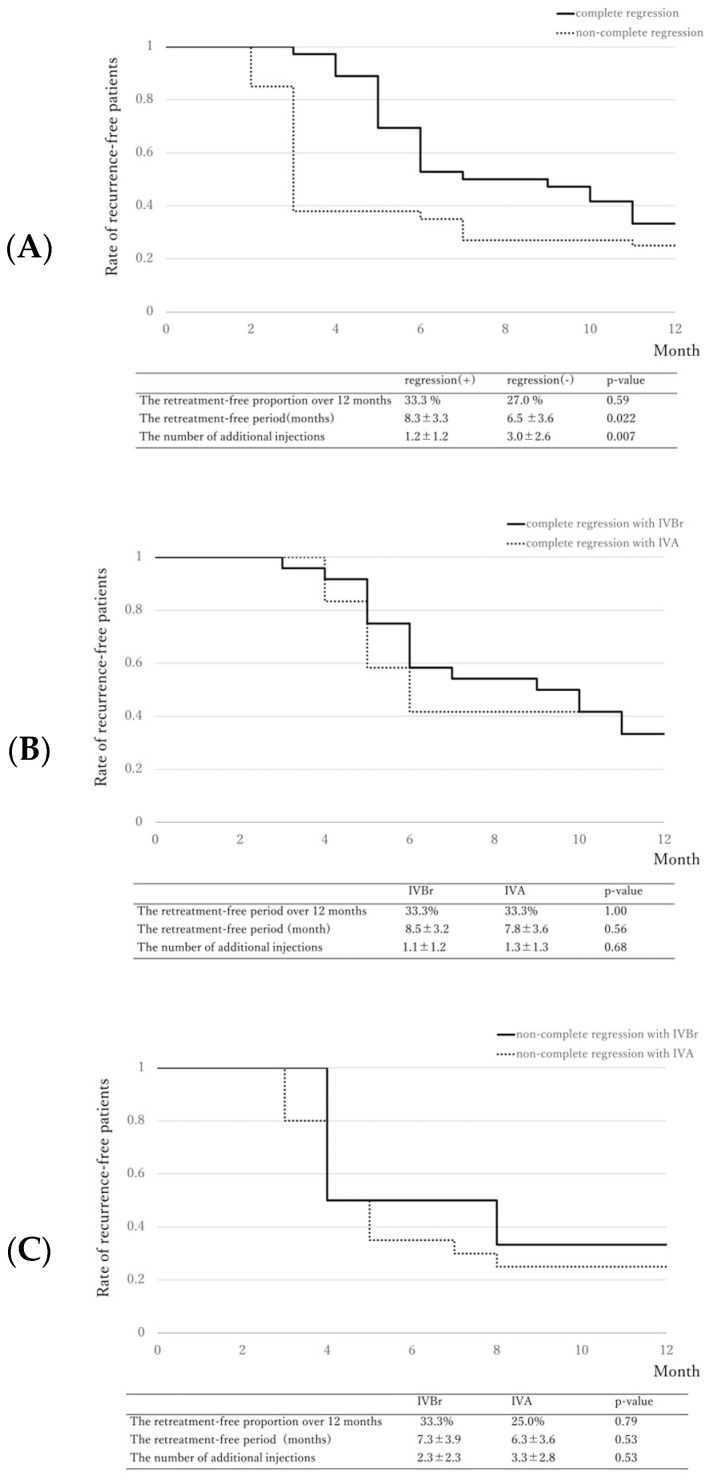
Kaplan–Meier survival curve associated with retreatment-free proportion. (**A**) Although the retreatment-free proportion between the complete regression group and the non-complete regression group was comparable (33.0% versus 27.0%, *p* = 0.59), the retreatment-free period was significantly longer in the complete regression group than in the non-complete regression group (8.3 ± 3.3 months versus 6.5 ± 3.6 months, *p* = 0.022), and the number of additional injections was significantly fewer in the complete regression group than in the non-complete regression group (1.2 ± 1.2 vs 3.0 ± 2.6, *p* = 0.007). (**B**) Comparing the complete regression group treated with brolucizumab or aflibercept, there were no significant differences in the retreatment-free proportion over 12 months, the retreatment-free period, and the number of additional injections between both groups. (**C**) Comparing the non-complete regression group treated with brolucizumab or aflibercept, there were no significant differences in the retreatment-free proportion over 12 months, the retreatment-free period, and the number of additional injections between the two groups.

Figure 2 shows the Kaplan–Meier curve associated with retreatment-free proportion after the loading injections.

## 3. Discussion

Currently, many reports on the response of PCV to treatment are available, including the effectiveness of anti-VEGF drugs as monotherapy or combined with PDT, its effect on subfoveal choroidal thickness, fellow eye conditions on treatment, and the presence or absence of choroidal vascular hyperpermeability which may predict treatment response [20,21,22,23]. In this study, we focused on the presence or absence of the complete regression of polyps after 3-monthly loading doses of brolucizumab or aflibercept. Although there were no significant differences in BCVA improvement between the complete regression group and the non-complete regression group at 12 months, there was a significant difference in the retreatment-free period and the number of additional injections needed between the two groups under the PRN regimen. Baseline smaller maximum polyp size and brolucizumab treatment were observed to be associated with the complete regression of polyp(s) at the third-month visit. The previous study reported that the complete regression of polyp(s) at 3 months is associated with better visual outcomes and a decreased number of additional injections at 12 months under a modified treat-and-extend regimen. 

In PCV, exudation is believed to be derived from polyps or branching vascular networks [24]. In the present study, the mean retreatment-free period after the loading phase was 8.3 months in the complete regression group, and the 12-month retreatment-free proportion was 33%, indicating that complete regression of polyps observed during the third-month visit may signify a longer interval before recurrence. However, it is important to note that recurrence can still occur even if complete regression was observed at the 3rd-month visit. In the non-complete regression group, the 12-month retreatment-free proportion at 27% was not significantly different from the complete regression group, indicating that some active polyps may still subside even if complete regression was not immediately achieved. Further studies on polyp characteristics that may lead to reactivation or regression are still warranted. 

Several studies investigated the factors predictive of the complete regression of polyp after treatment. The EVEREST study reported that the rate of complete polyp regression was significantly higher in the group treated with PDT and ranibizumab compared to the ranibizumab monotherapy group [5]. It also reported that a polyp with pulsation noted on ICGA was refractory to regression [25]. The exact reason as to why polyps with pulsations on ICGA were not likely to regress completely is still unclear. In a recent study by Choi et al., they observed that the complete filling time of a polyp was faster in eyes with pulsatile PCV than in non-pulsatile PCV [26]. Furthermore, PCV with polyp pulsation frequently caused hemorrhagic complications, and they speculated that pulsation could be detected when polyps are directly connected to the choroidal artery. Polyps with pulsations might be a distinct clinical characteristic from polyps without pulsation. 

In a study on PCV treated with ranibizumab monotherapy, a smaller maximum polyp diameter was associated with complete regression [27]. Although aflibercept and brolucizumab were used in our study, we also observed that the baseline maximum polyp diameter was associated with its regression, which further supports previous findings that baseline polyp size might be an important factor for complete regression. 

We observed that brolucizumab showed a higher rate of complete regression of polyps compared with aflibercept similar to previous studies. Brolucizumab has the smallest molecular weight (26 kDa) of the commercially available VEGF inhibitors, which enables it to administrate approximately 10 times greater molecular dose in a single injection compared with aflibercept [28]. This may explain why the complete regression rate is higher in brolucizumab than in aflibercept. However, brolucizumab causes more frequent intraocular inflammation compared with aflibercept [29]. In the HAWK and HARRIER, the incidence of IOI, IOI with vasculitis, and IOI with vasculitis and occlusion was 4.6%, 3.3%, and 2.1%, respectively [30].

This study was retrospective; hence, we are limited to associations, and causation could not be identified. The use of brolucizumab or aflibercept for treatment was also dependent on when these VEGF inhibitors were available in our institution. Another limitation is the small number of patients recruited in this study and a larger sample size is still necessary to further validate the present conclusions. If the future randomized controlled study confirms that the complete regression of polyps leads to a decreased number of additional injections, physicians will select various treatment regimens. 

We observed that the interval between treatment and recurrence is significantly longer in eyes with complete regression after the loading phase of VEGF inhibitors compared with eyes with non-complete regression, regardless of the type of VEGF inhibitor administered. Furthermore, the number of additional injections is significantly fewer in eyes with complete regression compared with non-complete regression eyes.

## 4. Materials and Methods

### 4.1. Patient Selection

A retrospective medical chart review was performed on 62 consecutive patients with PCV treated with intravitreal injection of either aflibercept (2.0 mg/0.05 mL) or brolucizumab (6.0 mg/0.05 mL) between April 2018 and April 2023 who fulfilled the following inclusion criteria: (1) treatment naïve eyes, (2) images of simultaneously-acquired OCT and ICGA at baseline (initial visit), 3-month visit were all available, (3) no other ophthalmic treatment received, including cataract surgery, (4) follow-up period of at least 12 months. The type of VEGF inhibitor was dependent on the time course—aflibercept was used between April 2018 and July 2020, while brolucizumab was used between August 2020 and April 2023. This retrospective study was approved by the Institutional Review Board of the University of Yamanashi (Approval No. 2676) and was conducted following the tenets of the Declaration of Helsinki. 

### 4.2. Follow-Up and Examination

**Figure 3:**
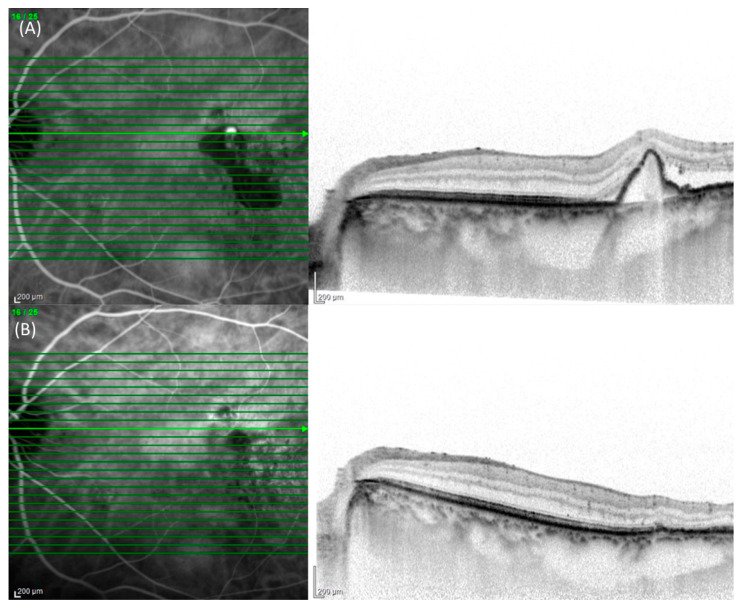
Indocyanine green angiography (ICGA) and cross-sectional spectral domain optical coherence tomography (SD-OCT) images of an 82-year-old female with complete regression of polyp after the loading phase of three monthly intravitreal brolucizumab administration. (**A**) At the baseline visit, ICGA showed hypercyanescence temporal to the macula corresponding to a protrusion of retinal pigment epithelium (RPE) with subretinal fluid (SRF) on SD-OCT. (**B**) One month after the loading phase, hypercyanescence disappeared on ICGA and a resolution of RPE protrusion and SRF was seen on SD-OCT.

**Figure 4:**
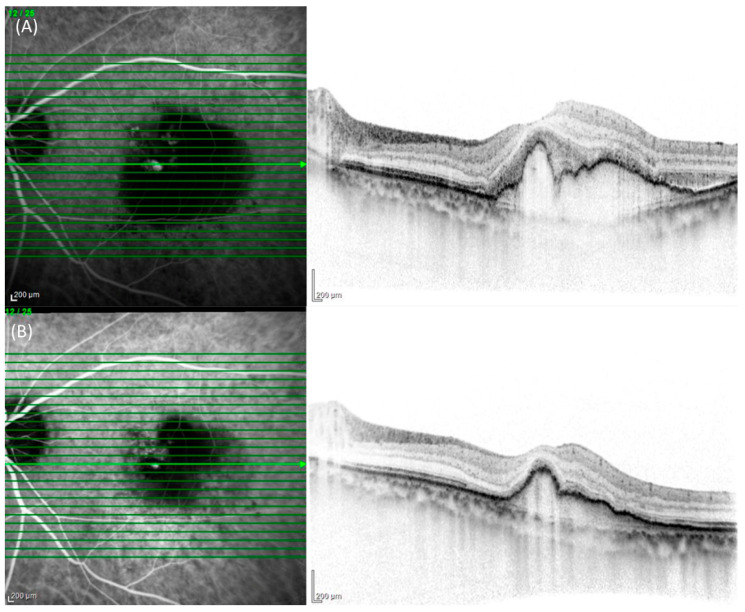
Indocyanine green angiography (ICGA) and cross-sectional spectral domain optical coherence tomography (SD-OCT) images of a 74-year-old female with partial regression of polyp after the loading phase of three monthly intravitreal brolucizumab administration. (**A**) At the baseline visit, ICGA showed hypercyanescence in the macula corresponding to RPE protrusion with subretinal hyperreflective material (SHRM) and SRF on SD-OCT. (**B**) One month after the loading phase, ICGA showed a smaller hypercyanescence lesion compared to the baseline. SD-OCT showed the resolution of exudation, including SHRM and SRF.

On initial consult, all patients underwent a comprehensive ophthalmic examination, which included measurement of the best corrected visual acuity (BCVA) using a Landolt chart, biomicroscopy with or without 78 diopter lens, measurement of intraocular pressure, color fundus photographs, fluorescein angiography (FA)/ICGA using a confocal laser scanning system (HRA2, Heidelberg Engineering, Germany), and SD-OCT (Spectralis Version 5.4 HRA + OCT, Heidelberg Engineering, Germany). All patients were followed up monthly and a comprehensive eye examination was performed at every visit. On the third month after the initial visit, aside from the comprehensive eye examination, FA/ICGA imaging was performed. The OCT scans were performed using 25 horizontal B-scans spanning 20° × 25°. Central retinal thickness was defined as the vertical distance between the inner limiting membrane and the surface of the retinal pigment epithelium (RPE), while the subfoveal choroidal thickness was the vertical distance between the chorioscleral border and the bottom of RPE on SD-OCT and both were measured manually. Maximum polyp size was also manually measured using the software’s built-in caliper on baseline ICGA images.(Ver 6, Heidelberg Engineering, Germany) The presence or absence of complete regression of polyp was independently judged by two senior ophthalmologists (MK and YS), and all eyes were classified into either the complete regression or non-complete regression group. Discordant gradings were resolved through open arbitration. Representative images of complete and non-complete regression are shown in Figure 3 and Figure 4, respectively.

### 4.3. Statistical Analysis

Statistical analysis was performed using SPSS version 28 (IBM, Tokyo, Japan). Categorical variables between the two groups were compared using a Chi-square test, and numerical variables between the two groups were compared using the Mann–Whitney U test. Similar values between the baseline and any visit were compared using a paired *t*-test. A *p*-value less than 0.05 was considered significant.

## Figures and Tables

**Table 1:** Clinical characteristics of patients with polypoidal choroidal vasculopathy.

Characteristics *n* = 62	Values
Age (years)	72.7 ± 7.9
Male (%)/Female (%)	47 (76)/15 (24)
BCVA (logMAR)	0.28 ± 0.28
CRT (μm)	320 ± 130.0
SCT (μm)	210 ± 90
Number of polyps	2.7 ± 2.6
Maximum diameter of polyps (μm)	362 ± 214

BCVA: best-corrected visual acuity, logMAR: logarithmic minimum angle of resolution, CRT: central retinal thickness, SCT: subfoveal choroidal thickness.

**Table 2:** Comparison of baseline characteristics between complete regression group and non-complete regression group.

Characteristics	Complete Regression Group (*n* = 36)	Non- Regression Group (*n* = 26)	*p*-Value	Adjusted *p*-Value
Age (years)	73.8 ± 7.7	71.1 ± 8.0	0.11	
Male (%)/Female (%)	29 (81)/7 (19)	18 (69)/8 (31)	0.31	
BCVA (logMAR)	0.29 ± 0.29	0.28 ± 0.28	0.86	
CRT (μm)	211 ± 96.0	208 ± 84	0.86	
SCT (μm)	304 ± 121	342 ± 142	0.21	
Number of polyps	2.5 ±2.5	3.0 ± 2.8	0.17	
Maximum diameter of polyps(μm)	293 ± 140	457 ± 262	0.024 *	0.005 *
IVBr (%)/IVA (%)	24 (67)/12 (33)	6 (23)/20 (77)	0.001 *	0.012 *

BCVA: best-corrected visual acuity, logMAR: logarithmic minimum angle of resolution, CRT: central retinal thickness, SCT: subfoveal choroidal thickness, IVBr: intravitreal brolucizumab, IVA: intravitreal aflibercept. The adjusted *p*-value was examined by multivariate analysis adjusting for age, gender, BCVA, CRT, SCT, and number of polyps. * means a *p*-value less than 0.05.

## Data Availability

The data presented in this study are available on request from the corresponding author. The data are not publicly available due to the prevention of the abuse.

## References

[1] Sakurada Y., Yoneyama S., Sugiyama A., Tanabe N., Kikushima W., Mabuchi F., Kume A., Kubota T., Iijima H. (2016). Prevalence and Genetic Characteristics of Geographic Atrophy among Elderly Japanese with Age-Related Macular Degeneration. PLoS ONE.

[2] Maruko I., Iida T., Saito M., Nagayama D., Saito K. (2007). Clinical characteristics of exudative age-related macular degeneration in Japanese patients. Am. J. Ophthalmol..

[3] Yamashiro K., Yanagi Y., Koizumi H., Matsumoto H., Cheung C.M.G., Gomi F., Iida T., Tsujikawa A. (2022). Relationship between Pachychoroid and Polypoidal Choroidal Vasculopathy. J. Clin. Med..

[4] Sakurada Y., Tanaka K., Fragiotta S. (2023). Differentiating drusen and drusenoid deposits subtypes on multimodal imaging and risk of advanced age-related macular degeneration. Jpn. J. Ophthalmol..

[5] Koh A., Lee W.K., Chen L.J., Chen S.J., Hashad Y., Kim H., Lai T.Y., Pilz S., Ruamviboonsuk P., Tokaji E. (2012). EVEREST study: Efficacy and safety of verteporfin photodynamic therapy in combination with ranibizumab or alone versus ranibizumab monotherapy in patients with symptomatic macular polypoidal choroidal vasculopathy. Retina.

[6] Lee W.K., Iida T., Ogura Y., Chen S.J., Wong T.Y., Mitchell P., Cheung G.C.M., Zhang Z., Leal S., Ishibashi T. (2018). Efficacy and Safety of Intravitreal Aflibercept for Polypoidal Choroidal Vasculopathy in the PLANET Study: A Randomized Clinical Trial. JAMA Ophthalmol..

[7] Matsumoto H., Hoshino J., Mukai R., Nakamura K., Akiyama H. (2021). Short-term outcomes of intravitreal brolucizumab for treatment-naive neovascular age-related macular degeneration with type 1 choroidal neovascularization including polypoidal choroidal vasculopathy. Sci. Rep..

[8] Matsumiya W., Honda S., Otsuka K., Miki A., Nagai T., Imai H., Kusuhara S., Nakamura M. (2017). One-year outcome of combination therapy with intravitreal aflibercept and verteporfin photodynamic therapy for polypoidal choroidal vasculopathy. Graefe’s Arch. Clin. Exp. Ophthalmol..

[9] Morimoto M., Matsumoto H., Mimura K., Akiyama H. (2017). Two-year results of a treat-and-extend regimen with aflibercept for polypoidal choroidal vasculopathy. Graefe’s Arch. Clin. Exp. Ophthalmol..

[10] Kikushima W., Sakurada Y., Sugiyama A., Tanabe N., Kume A., Iijima H. (2017). Comparison of initial treatment between 3-monthly intravitreal aflibercept monotherapy and combined photodynamic therapy with single intravitreal aflibercept for polypoidal choroidal vasculopathy. Graefe’s Arch. Clin. Exp. Ophthalmol..

[11] Fukuda Y., Sakurada Y., Matsubara M., Hasebe Y., Sugiyama A., Kikushima W., Kashiwagi K. (2021). Comparison of Outcomes between 3 Monthly Brolucizumab and Aflibercept Injections for Polypoidal Choroidal Vasculopathy. Biomedicines.

[12] Ito A., Maruyama-Inoue M., Kitajima Y., Ikeda S., Inoue T., Kadonosono K. (2022). One-year outcomes of intravitreal brolucizumab injections in patients with polypoidal choroidal vasculopathy. Sci. Rep..

[13] Ijiri S., Sugiyama K. (2015). Short-term efficacy of intravitreal aflibercept for patients with treatment-naive polypoidal choroidal vasculopathy. Graefe’s Arch. Clin. Exp. Ophthalmol..

[14] Tanaka K., Koizumi H., Tamashiro T., Itagaki K., Nakayama M., Maruko I., Wakugawa S., Terao N., Onoe H., Wakatsuki Y. (2022). Short-term results for brolucizumab in treatment-naive neovascular age-related macular degeneration: A Japanese multicenter study. Jpn. J. Ophthalmol..

[15] Fukuda Y., Sakurada Y., Matsubara M., Kotoda Y., Kasai Y., Sugiyama A., Kashiwagi K. (2023). Comparison of one-year outcomes between as-needed brolucizumab and aflibercept for polypoidal choroidal vasculopathy. Jpn. J. Ophthalmol..

[16] Azzolini C., Pagani I.S., Pirrone C., Borroni D., Donati S., Al Oum M., Pigni D., Chiaravalli A.M., Vinciguerra R., Simonelli F. (2013). Expression of VEGF-A, Otx homeobox and p53 family genes in proliferative vitreoretinopathy. Mediat. Inflamm..

[17] Yamamoto A., Okada A.A., Kano M., Koizumi H., Saito M., Maruko I., Sekiryu T., Iida T. (2015). One-Year Results of Intravitreal Aflibercept for Polypoidal Choroidal Vasculopathy. Ophthalmology.

[18] Sayanagi K., Fujimoto S., Hara C., Fukushima Y., Maruyama K., Kawasaki R., Sato S., Nishida K. (2024). Effect of polyp regression and reduction on treatment efficacy in polypoidal choroidal vasculopathy treated with aflibercept. Sci. Rep..

[19] Borroni D., Erts R., Vallabh N.A., Bonzano C., Sepetiene S., Krumina Z., Romano V., Parekh M., Iannetta D. (2021). Solar retinopathy: A new setting of red, green, and blue channels. Eur. J. Ophthalmol..

[20] Matsubara M., Sakurada Y., Sugiyama A., Fukuda Y., Parikh R., Kashiwagi K. (2020). Response to photodynamic therapy combined with intravitreal aflibercept for polypoidal choroidal vasculopathy depending on fellow-eye condition:2-year results. PLoS ONE.

[21] Fukuda Y., Sakurada Y., Sugiyama A., Yoneyama S., Matsubara M., Kikushima W., Tanabe N., Parikh R., Kashiwagi K. (2020). Pachydrusen in Fellow Eyes Predict Response to Aflibercept Monotherapy in Patients with Polypoidal Choroidal Vasculopathy. J. Clin. Med..

[22] Yanagi Y., Ting D.S.W., Ng W.Y., Lee S.Y., Mathur R., Chan C.M., Yeo I., Wong T.Y., Cheung G.C.M. (2018). Choroidal Vascular Hyperpermeability as a Predictor of Treatment Response for Polypoidal Choroidal Vasculopathy. Retina.

[23] Sakurada Y., Sugiyama A., Tanabe N., Kikushima W., Kume A., Iijima H. (2017). Choroidal Thickness as a Prognostic Factor of Photodynamic Therapy with Aflibercept or Ranibizumab for Polypoidal Choroidal Vasculopathy. Retina.

[24] Dansingani K.K., Gal-Or O., Sadda S.R., Yannuzzi L.A., Freund K.B. (2018). Understanding aneurysmal type 1 neovascularization (polypoidal choroidal vasculopathy): A lesson in the taxonomy of ‘expanded spectra’-A review. Clin. Exp. Ophthalmol..

[25] Tan C.S., Cheung C.M., Lai T.Y., Pataluskaite R., Margaron P., Lim T.H. (2022). Predictors and importance of complete polypoidal lesion regression in the Everest II study: predictors of polyp regression in polypoidal choroidal vasculopathy. Retina.

[26] Choi K.E., Lee Y.J., Bae S.H. (2024). Imaging and clinical features of pulsatile polypoidal choroidal vasculopathy. Retina.

[27] Koizumi H., Yamagishi T., Yamazaki T., Kinoshita S. (2011). Predictive factors of resolved retinal fluid after intravitreal ranibizumab for polypoidal choroidal vasculopathy. Br. J. Ophthalmol..

[28] Nguyen Q.D., Das A., Do D.V., Dugel P.U., Gomes A., Holz F.G., Koh A., Pan C.K., Sepah Y.J., Patel N. (2020). Brolucizumab: Evolution through Preclinical and Clinical Studies and the Implications for the Management of Neovascular Age-Related Macular Degeneration. Ophthalmology.

[29] Dugel P.U., Koh A., Ogura Y., Jaffe G.J., Schmidt-Erfurth U., Brown D.M., Gomes A.V., Warburton J., Weichselberger A., Holz F.G. (2020). HAWK and HARRIER: Phase 3, Multicenter, Randomized, Double-Masked Trials of Brolucizumab for Neovascular Age-Related Macular Degeneration. Ophthalmology.

[30] Mones J., Srivastava S.K., Jaffe G.J., Tadayoni R., Albini T.A., Kaiser P.K., Holz F.G., Korobelnik J.F., Kim I.K., Pruente C. (2021). Risk of Inflammation, Retinal Vasculitis, and Retinal Occlusion-Related Events with Brolucizumab: Post Hoc Review of HAWK and HARRIER. Ophthalmology.

